# Peripheral blood circMAN1A2 as a novel diagnostic biomarker for gout: integrated transcriptomic analysis and clinical validation

**DOI:** 10.3389/fmolb.2026.1791371

**Published:** 2026-05-26

**Authors:** Wenyan Guo, Kaixi Liu, Jianhong Sun, Qiaoxin Zhang, Xiaoyang Jiao, Peiting Ru, Yanrong Chen, Xinran Yang

**Affiliations:** 1 The Department of Clinical Laboratory, General Hospital of Southern Theater Command, Guangzhou, China; 2 The Department of Clinical Laboratory, Shantou Central Hospital, Shantou, China; 3 The Department of Clinical Laboratory, The First Affiliated Hospital of Shantou University Medical College, Shantou, China; 4 Department of Cell Biology and Genetics, Medical College of Shantou University, Shantou, China; 5 The Department of Clinical Laboratory, Guangzhou Nansha District Hospital of Traditional Chinese Medicine, Guangzhou, China

**Keywords:** bioinformatics, circMAN1A2, circRNA, gout, molecular diagnostics

## Abstract

**Background:**

Gout is one of the most common inflammatory arthritides. Although identification of monosodium urate crystals in synovial fluid is the diagnostic gold standard, the invasive procedure limits routine use. Non-invasive biomarkers are needed to facilitate timely diagnosis of acute gout flares and to differentiate gout from asymptomatic hyperuricaemia

**Methods:**

Differential expression analysis was performed using the GEO circRNA microarray dataset GSE178825 (peripheral blood mononuclear cells, PBMCs; gout vs. healthy controls). Differentially expressed circRNAs were mapped to their host genes, followed by host-gene-based functional enrichment and protein–protein interaction (PPI) network analyses for candidate prioritization, circMAN1A2, derived from the MAN1A2 locus, was selected for clinical validation. PBMCs were isolated from whole blood samples obtained from 30 patients with acute gout flare, 30 individuals with HUA, and 30 healthy controls. Circularity was confirmed by RNase R resistance, and circMAN1A2 expression was quantified by RT-qPCR. Diagnostic performance was evaluated using receiver operating characteristic (ROC) analysis.

**Results:**

Peripheral blood circMAN1A2 levels were significantly lower in patients with gout than in healthy controls, and showed a decreasing trend compared with individuals with HUA. circMAN1A2 showed preliminary diagnostic potential for gout (AUC = 0.86; 95% CI 0.66–1.00), with 90.0% sensitivity and 90.1% specificity.

**Conclusion:**

Peripheral blood circMAN1A2 is a promising candidate biomarker for gout diagnosis. Integrating public transcriptomic mining with targeted clinical validation may accelerate biomarker discovery for gout.

## 1 Introduction

Gout is the leading cause of inflammatory arthritis worldwide, with incidence rising globally (Dalbeth et al., 2021). It arises from disordered purine metabolism, leading to hyperuricaemia and subsequent deposition of monosodium urate (MSU) crystals (Yip and Berman, 2021; Singh and Gaffo, 2020). Acute flares cause severe joint pain, while chronic crystal deposition drives irreversible joint damage (Clebak et al., 2020; Xu et al., 2023). Beyond musculoskeletal complications, gout is also associated with renal dysfunction (Yang et al., 2018; Tu et al., 2023; Ya et al., 2021), cardiovascular disease (Anderson and Knowlton, 2022), and increased mortality, underscoring the need for early intervention (Bardin and Richette, 2017; Peng et al., 2023). However, misdiagnosis and undertreatment remain prevalent, partly due to limitations in current diagnostic tools (Du et al., 2024). A major clinical challenge is distinguishing acute gout from asymptomatic hyperuricaemia (HUA), as elevated serum uric acid does not necessarily indicate gout and many individuals with HUA never develop clinical symptoms. This distinction is particularly important when invasive procedures are unavailable or impractical (Mandl et al., 2023). Therefore, minimally invasive, peripheral blood-based biomarkers are urgently needed to improve diagnostic accuracy and guide timely management.

Current diagnostic pathways rely on MSU crystal identification *via* synovial fluid analysis, the 2015 ACR/EULAR gold standard (FitzGerald et al., 2020; Neogi et al., 2015). However, this procedure is invasive, technically dependent, and often avoided in routine practice or acute settings. Imaging modalities like dual-energy computed tomography (DECT) or ultrasonography visualize urate deposits but exhibit limited sensitivity in early disease and are not universally accessible (Dalbeth et al., 2019; Lee et al., 2020). Serum uric acid (SUA), the most widely used surrogate marker, also has limited diagnostic utility. Up to 40% of acute gout patients may present with normal SUA levels, whereas many individuals with HUA remain asymptomatic and never develop gout (Lee et al., 2020). Expert guidelines therefore caution against diagnosing gout based solely on SUA levels (Mandl et al., 2023). These limitations create a pressing clinical need for alternative biomarkers that are non-invasive, accessible, and capable of distinguishing gout from asymptomatic HUA with greater diagnostic precision.

Gout pathogenesis involves interplay between genetic susceptibility and dysregulated inflammatory responses (Huang et al., 2020; Zhong et al., 2021). Immune cells in peripheral blood play a central role in MSU crystal recognition and cytokine release including IL-1β, IL-6, TNF-α) (Galozzi et al., 2021), suggesting that transcriptional changes in these cells could serve as disease-specific signals. Circular RNAs (circRNAs)—covalently closed, stable transcripts—are emerging as ideal candidates for such biomarkers. Their resistance to degradation enables robust detection in peripheral blood mononuclear cells (PBMCs), and their cell-type-specific expression may reflect immune activation states (Chen et al., 2021; Panda, 2018; Najafi, 2023; Zhou et al., 2020). Bioinformatic mining of public transcriptomic datasets further accelerates discovery by prioritizing candidates for clinical validation (Z et al., 2023; Bian et al., 2021). While circRNAs show promise in rheumatic diseases like rheumatoid arthritis (Cai et al., 2021; Yang et al., 2020; Zhang et al., 2025), the circRNA landscape in gout remains underexplored, with only one prior study proposing hsa_circRNA_102911 based on pyroptosis pathways (Niu et al., 2023). Comprehensive screening for PBMC-derived circRNAs with validated diagnostic performance, particularly for distinguishing gout from HUA, remains lacking.

Here, we employed a discovery-to-validation pipeline to identify PBMC-derived circRNA biomarkers for gout. Using the GEO microarray dataset GSE178825, we first screened differentially expressed circRNAs between gout patients and healthy controls. To prioritize functionally relevant candidates, we mapped circRNAs to host genes, performed enrichment analyses, and constructed protein–protein interaction (PPI) networks to identify hub genes. MAN1A2 emerged as a top candidate, and its cognate circRNA (circMAN1A2) was selected for validation. We measured circMAN1A2 expression in PBMCs from three cohorts: acute gout flares, HUA, and healthy controls. Our primary goal was to evaluate circMAN1A2’s ability to distinguish gout from both healthy controls and HUA—a critical clinical distinction—and assess its potential as a clinically translatable biomarker compatible with routine qPCR-based workflows.

## 2 Materials and methods

### 2.1 Data selection

The circRNA dataset GSE178825 was downloaded from GEO (https://www.ncbi.nlm.nih.gov/geo/). Expression data were generated on platform GPL21825 (Arraystar Human circRNA microarray V2). The dataset includes five gout samples and three healthy control samples.

### 2.2 Data processing

Raw data were processed in R using the limma package. Sample quality was assessed, and samples were grouped as gout (n = 5) and controls (n = 3) based on the clinical annotations. Data were normalized by probe/gene ID. Differentially expressed genes (DEGs) were identified using |log2 fold change| > 1 and adjusted *p* < 0.05.

### 2.3 Functional enrichment analysis

Gene Ontology (GO) enrichment was performed with DAVID (https://david.ncifcrf.gov/) and visualized in R. Kyoto Encyclopedia of Genes and Genomes (KEGG) pathway enrichment was conducted using OmicShare (https://www.omicshare.com/tools/). A threshold of *p* < 0.05 was used for significance.

### 2.4 Construction of PPI network and hub gene identification

PPI among DEGs were queried in STRING *v*11.5 (https://cn.string-db.org/) with default settings. The network was visualized in Cytoscape 3.9.1. Hub DEGs were ranked with cytoHubba using Density of Maximum Neighborhood Component (DMNC) (local) and Eccentricity (EC) (global) metrics, and the top 25 were selected.

### 2.5 Patients and sample collection

Due to the significantly higher prevalence of gout in the male population at our clinical center during the study period, our validation cohort was restricted to male participants. Specifically, Male patients with idiopathic gout (Crystal-associated arthropathy caused by MSU deposition (Dalbeth et al., 2019)), individuals with primary hyperuricemia, and healthy volunteers were recruited at the First Affiliated Hospital of Shantou University Medical College (Shantou, China) from June to October 2022. Inclusion criteria: (1) gout diagnosed by clinical features with evidence of MSU deposition by ultrasonography or laboratory examination; (2) hyperuricemia defined as no clinical manifestations of gout and serum uric acid ≥428 μmol/L (Wang et al., 2022). Exclusion criteria: malignancy, tuberculosis, hepatic or renal insufficiency, or autoimmune disease. Demographics and laboratory data (age, sex, TC, TG, LDL, HDL, UA, Cr, GLU, CRP) were collected. Written informed consent was obtained from all participants. The study was approved by the Ethics Committee of the First Affiliated Hospital of Shantou University Medical College.

### 2.6 PBMC isolation, RNA extraction, and quantitative RT–PCR

Peripheral blood samples were collected into anticoagulant tubes. PBMCs were isolated by Ficoll density-gradient centrifugation. Total RNA was extracted from PBMCs using the UNIQ-10 TRIzol kit (Sangon Biotech, Shanghai, China). RNA integrity was assessed by denaturing agarose gel electrophoresis; concentration and purity were measured with a NanoDrop 2000. cDNA was synthesized with Maxima Reverse Transcriptase (Thermo Fisher Scientific, Waltham, MA). qPCR was performed on aQuantStudio™1 Plus Fluorescence Quantitative PCR (ABI/Thermo Fisher, United States of America) using SYBR chemistry and 2x SG Fast qPCR Master Mix (High Rox) (Sangon Biotech, Shanghai, China). Cycling conditions: 95 °C for 3 min (1 cycle), 45 cycles of 95 °C for 15 s and 60 °C for 30 s. Relative expression of circMAN1A2 was calculated by the 2^−(ΔΔCt)^ method.

### 2.7 Statistical analyses

Data are presented as mean ± SD. Group differences were assessed with the Kruskal–Wallis test followed by Bonferroni-corrected pairwise comparisons. Spearman correlation evaluated associations between clinical variables and relative MAN1A2 expression. Diagnostic performance was assessed by ROC curves; AUCs were compared with DeLong’s test, and sensitivities/specificities with McNemar’s test. Analyses were performed in SPSS 20.0. Two-sided *p* < 0.05 was considered statistically significant.

## 3 Results

### 3.1 Identification and functional enrichment analysis of differentially expressed genes

**FIGURE 1:**
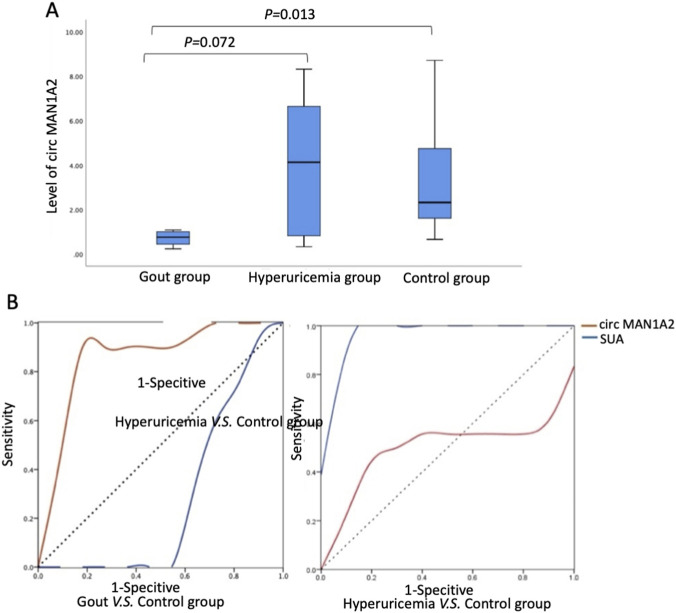
Differentially expressed genes and enrichment analysis in gout. **(A)** Volcano map of DEGs. **(B)** Heat map of DGEs (colour from blue to red represents circRNA expression from low to high. Each column in the map represents a sample, and each row represents a gene). **(C)** The result of GO of DEGs. **(D,E)** The result of GO of DEGs and top 25 of KEGG enrichment).

Differential expression analysis between patients with gout and healthy controls identified 56 differentially expressed genes (DEGs) among 4,994 detected genes, including 43 upregulated and 13 downregulated genes (|log_2_FC| > 1, adjusted *P* < 0.05). The distribution and clustering of DEGs are displayed in the integrated volcano plot and heatmap, respectively (Figures 1A,B). To explore the biological functions of these DEGs, Gene Ontology (GO) and Kyoto Encyclopedia of Genes and Genomes (KEGG) enrichment analyses were performed. In total, 144 GO terms were significantly enriched, including 28 biological process (BP) terms, 91 cellular component (CC) terms, and 25 molecular function (MF) terms (Table 1). GO enrichment highlighted processes related to DNA repair, sphingolipid biosynthetic process, response to hydrogen peroxide, and protein glycosylation (BP); cytosol, nucleus, nucleoplasm, and membrane (CC); and ATP binding and DNA binding (MF) (Figure 1C). KEGG enrichment analysis indicated that DEGs were mainly enriched in pathways related to sphingolipid metabolism, Hedgehog signaling pathway, choline metabolism in cancer, *Yersinia* infection, and cell cycle, suggesting potential links between metabolic/signaling dysregulation and gout pathophysiology (Figures 1D,E).

**TABLE 1:** The result of GO of DGEs.

Category	Term	Count	*P* value	Genes
CC	Cytosol	27	8.22E-05	USP36, MTPN, SAMD8, CHD9, GSTP1, ANKRD12, SKA3, RPL9, BACH1, FGD4, GPBP1, CPNE1, PIP5K1A, SHOC2, PRKG1, SPECC1, JUN, MBNL1, ANAPC7, NEK6, SMARCA5, TTLL5, PRKD3, APLF, RGS12, PICALM, CSNK1G1
CC	Nucleoplasm	21	0.00040114	SPECC1, USP36, SHPRH, JUN, MBNL1, ANAPC7, CHD9, NONO, NEK6, ANKRD12, SMARCA5, BACH1, SLC8A1, CDC14B, PRKD3, APLF, CPNE1, PIP5K1A, SHOC2, RGS12, RPRD1A
CC	Nucleus	24	0.005756498	USP36, CSNK1G3, MTPN, JUN, MBNL1, ANAPC7, SGMS1, GSTP1, NONO, NEK6, SMARCA5, RPL9, BACH1, CDC14B, TTLL5, GPBP1, APLF, CPNE1, PIP5K1A, SHOC2, RGS12, MALAT1, PICALM, CSNK1G1
CC	Membrance	13	0.014969822	SPECC1, SGMS1, NONO, RPL9, SLC8A1, RSL1D1, PRKD3, **MAN1A2**, CPNE1, DYM, ME2, PICALM, PCMTD1
CC	RNA polymerase II transcription factor complex	3	0.034013155	JUN, NONO, BACH1
CC	Fibrillar center	3	0.049533356	SPECC1, NONO, SMARCA5
BP	Negative regulation of vascular smooth muscle cell proliferation	3	0.005856712	GSTP1, SOD2, PRKG1
BP	DNA repair	5	0.006577362	SHPRH, NONO, SMARCA5, BACH1, CDC14B
BP	Sphingolipid biosynthetic process	3	0.006912223	SAMD8, SGMS1, PRKD3
BP	Hydrogen peroxide reaction	3	0.008047357	JUN, SOD2, SLC8A1
BP	Response to L-ascorbic acid	2	0.017679957	GSTP1, SOD2
BP	Sphingomyelin biosynthesis	2	0.027644927	SAMD8, SGMS1
BP	Regulation of cellular senescence	2	0.030120655	RSL1D1, NEK6
BP	Protein glycosylation	3	0.046314031	MAN1A2, B4GALT6, UGGT2
BP	Response to muscle stretch	2	0.049705441	JUN, SLC8A1
MF	Nucleosome-dependent ATPase activity	3	0.003257537	SHPRH, CHD9, SMARCA5
MF	Ceramide cholinephosphotransferase activity	2	0.007477298	SAMD8, SGMS1
MF	Ceramide phosphoethanolamine synthase activity	2	0.007477298	SAMD8, SGMS1
MF	Sphingomyelin synthase activity	2	0.007477298	SAMD8, SGMS1
MF	ATP binding	10	0.013033894	CSNK1G3, SHPRH, TTLL5, PRKD3, CHD9, NEK6, SMARCA5, PIP5K1A, PRKG1, CSNK1G1
MF	DNA binding	9	0.013485132	SHPRH, JUN, ZMYM4, CHD9, NONO, GPBP1, SMARCA5, SOD2, BACH1

### 3.2 Identification of hub genes and circMAN1A2-related regulatory network

**FIGURE 2:**
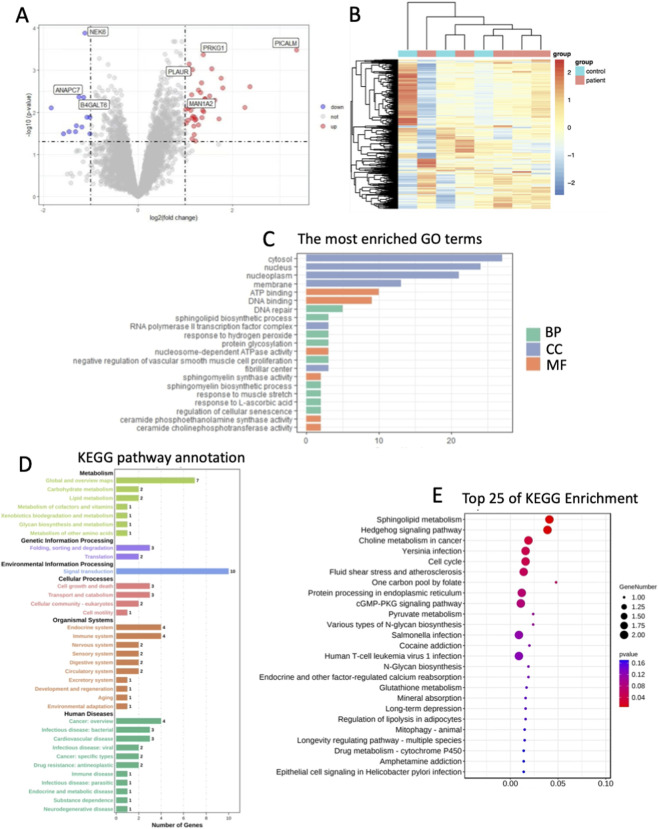
PPI network and circMAN1A2-related regulatory analysis. **(A)** PPI network of 56 DEGs constructed by STRING. **(B,C)** Top 25 hub genes ranked by DMNC and EC. **(D)** Predicted circMAN1A2–miRNA interactions and target gene MAN1A2 (Aggregate PCT = 0.36).

A total of 56 differentially expressed genes (DEGs) were imported into the STRING database to obtain PPI data, and the network was visualized using Cytoscape 3.9.1 (Figure 2A). Hub genes were ranked using the DMNC and EC algorithms, with 25 genes identified by each method and 23 overlapping candidates obtained, including GSTP1, CHD9, SRGN, ZMYM4, ANKRD12, NONO, MTPN, NUP54, TTLL5, MBNL1, ANAPC7, SOD2, MAN1A2, CDC14B, NEK6, PRKG1, RPL9, SLC8A1, SPECC1, LU2P6, JUN, and UGGT2 (Figures 2B,C). Bioinformatic analysis revealed that MAN1A2 was upregulated among DEGs associated with gout and correlated with pathways highlighted in enrichment analyses, indicating its centrality in the PPI network. GO and KEGG results further suggested that MAN1A2 is localized to the membrane and participates in glycoprotein biosynthesis.

Predicted interactions between circMAN1A2 (hsa_circMAN1A2_011) and microRNAs were analyzed using the circBank database and TargetScan 8.0, with Aggregate PCT >0.1 as the selection threshold. Three miRNAs, namely, hsa-miR-1297, hsa-miR-4465, and hsa-miR-26b-5p, were predicted to bind circMAN1A2, and MAN1A2 was identified as a potential target gene, with an Aggregate PCT value of 0.36 (Figure 2D).

### 3.3 Clinical validation and diagnostic value of circMAN1A2 in gout

Through gene ID conversion, hsa_circMAN1A2_011 was identified as the corresponding transcript of circRNA, hereafter referred to as circMAN1A2 in this study. To evaluate clinical relevance, circMAN1A2 expression in PBMCs was measured by RT–qPCR in 90 male participants including gout, hyperuricemia (HUA), and healthy control groups. Baseline clinical characteristics are summarized in Table 2. Age and total cholesterol did not differ significantly among the three groups. SUA levels were higher in the gout and HUA groups than in controls, but did not significantly differ between gout and HUA. Several metabolic and inflammatory parameters, including triglycerides, creatinine, glucose, CRP, LDL, and HDL, showed group-specific differences.

**TABLE 2:** Comparison of datas of patients in three groups (*P Value vs. control).

Indicator (median)	Gout (n = 30)	Hyperuricaemia (n = 30)	Control (n = 30)	*P* value
Age (years)	39.00 ± 3.60	34.25 ± 3.95	36.75 ± 2.22	0.267
TC (mmol/L)	4.45 ± 1.17	5.71 ± 1.03	4.56 ± 0.32	0.064
TG (mmol/L)	1.75 ± 1.04*	1.51 ± 1.03	0.90 ± 0.32	0.032
LDL (mmol/L)	3.09 ± 0.84	3.70 ± 0.82*	2.76 ± 0.53	0.043
HDL (mmol/L)	1.00 ± 0.12	1.41 ± 0.32*	1.34 ± 0.03	0.003
UA (μmol/L)	526.20 ± 176.44	557.74 ± 139.54*	378.40 ± 37.98	0.006
Cr (μmol/L)	121.32 ± 15.83*	87.41 ± 15.97	90.09 ± 11.58	<0.001
Glu (mmol/L)	5.20 ± 0.55*	5.18 ± 0.83*	4.26 ± 0.47	0.001
CRP (mg/L)	18.15 ± 41.81*	17.36 ± 24.58*	7.50 ± 0.98	0.004
MAN1A2 (2^−(ΔΔCt)^ )	0.69 ± 0.31*	3.95 ± 3.39	3.15 ± 2.42	0.010

Data are presented as mean ± SD., Differences among groups were assessed using one-way ANOVA, or Kruskal–Wallis test, as appropriate.

*: *P* Value <0.05 was considered to denote statistical significance.

**FIGURE 3:**
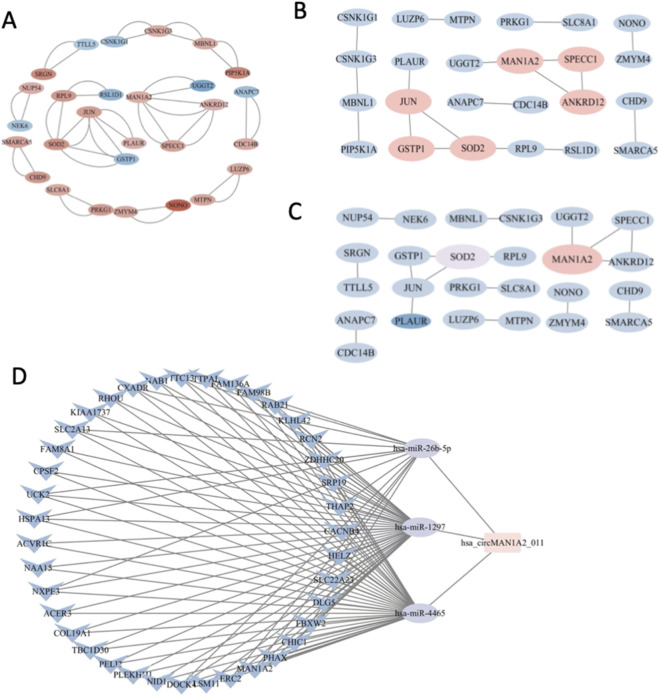
circMAN1A2 expression and diagnostic ROC analyses in gout and hyperuricemia. **(A)** circMAN1A2 expression levels in gout (n = 30), HUA (n = 30), and control groups (n = 30) quantified by the 2^−(ΔΔCt)^. **(B)** The ROC curve data of circMAN1A2 and UA.

The expression level of circMAN1A2 was significantly lower in the gout group compared with controls (*P* = 0.010) (Figure 3A). Correlation analysis (Table 3) revealed that blood glucose and creatinine levels were negatively correlated with relative circMAN1A2 expression (R = −0.400, *P* = 0.031; R = −0.484, *P* = 0.008), whereas high-density lipoprotein showed a positive correlation (R = 0.526, *P* = 0.003). In contrast, low-density lipoprotein, cholesterol, and triglycerides tended to be negatively correlated with MAN1A2 expression, although these associations did not reach statistical significance (*P* > 0.05).

**TABLE 3:** The correlation between each factor and circMAN1A2 (*P Value).

Variable	Total cholesterol (mmol/L)	Triglyceride (mmol/L)	Low‐density lipoprotein (mmol/L)	High‐density lipoprotein (mmol/L)	Uric acid (μmol/L)	Creatinine (μmol/L)	Blood glucose (mmol/L)	CRP (mg/L)
MAN1A2 (2^−(ΔΔCt)^ )	−0.189	−0.244	−0.286	0.526*	−0.216	−0.484*	−0.400*	−0.363
*P* value	0.326	0.202	0.133	0.003	0.260	0.008	0.031	0.063

Receiver operating characteristic (ROC) curve analysis was performed to evaluate the diagnostic performance of circMAN1A2 and SUA. For distinguishing gout from healthy controls, circMAN1A2 showed good diagnostic performance, with an area under the curve (AUC) of 0.86 (95% CI: 0.66–1.00). In contrast, SUA showed a low AUC of 0.28 (95% CI: 0.04–0.52), which may be partly explained by the known fluctuation or normalization of SUA during acute gout flares, thereby limiting its diagnostic value in this setting. For distinguishing HUA from healthy controls, circMAN1A2 showed limited diagnostic performance, with an AUC of 0.49 (95% CI: 0.20–0.79), whereas SUA showed excellent performance, with an AUC of 0.98 (95% CI: 0.92–1.00) (Table 4; Figure 3B). These results suggest that circMAN1A2 may be more informative for identifying gout-related inflammatory status than for detecting hyperuricemia alone.

**TABLE 4:** Diagnose efficiency of circMAN1A2 in differentiating the gout or hyperuricemia from normal control.

Test result Variable(s)	AUC(95%CI)	*P* value	SD	Cut-off point	Sensitivity (%)	Specificity (%)	PPV (%)	NPV (%)	LR+	LR-
Gout group *vs.* Control group										
circMAN1A2	0.86 (0.66,1.00)	0.006	0.10	1.22	90.0	90.1	89.0	91.0	9.89	0.11
SUA	0.28 (0.04,0.52)	0.091	0.12	276.50	100.0	9.10	50.0	100.0	1.10	0.00
Hyperuricemia group *vs.* Control group										
circMAN1A2	0.49 (0.20,0.79)	0.940	0.15	5.73	44.4	90.0	80.0	64.0	3.33	1.62
SUA	0.98 (0.92,1.00)	<0.001	0.03	411.47	100.0	90.0	90.0	100.0	8.89	0.00

## 4 Discussion

### 4.1 Pathophysiological basis of gout and gene identification

Gout is a chronic inflammatory disease caused by the deposition of monosodium urate (MSU) crystals in joints and tissues, and its incidence and prevalence are increasing worldwide (Dalbeth et al., 2019). Epidemiological studies have demonstrated that gout is closely associated with hyperuricemia, hyperlipidemia, obesity, and metabolic syndrome, all of which seriously endanger human health (Dalbeth et al., 2019; Dalbeth et al., 2016). Currently, the diagnosis of gout mainly relies on clinical manifestations and detection of MSU crystals (Bardin and Richette, 2017). However, these methods are limited by invasiveness, delayed detection, and suboptimal diagnostic performance. Therefore, identifying novel molecular markers to facilitate early and accurate diagnosis is urgently needed.

In this study, data-mining analysis identified differentially expressed genes (DEGs) between patients with gout and healthy controls. PICALM, PRKG1, MAN1A2, and PLAUR were upregulated DEGs, while NEK6, ANAPC7, and B4GALT6 were downregulated. PICALM is a clathrin-adapter protein critical to clathrin-mediated endocytosis and autophagy (Ando et al., 2022) and has been proposed as a cerebrospinal fluid biomarker for Alzheimer’s disease (Schjeide et al., 2011). PRKG1 encodes protein kinases essential for smooth muscle contractility (Micale et al., 2022). MAN1A2 (Mannosidase-1-Alpha-2), located in the membrane, participates in the upregulation of protein glycosylation (Jin et al., 2018). PLAUR serves as the receptor for Serpin E1 (Chen et al., 2022). Conversely, NEK6 functions as an injury-responsive kinase acting with STAT3 in reactive astrogliosis (Yu et al., 2022). ANAPC7 is a component of the anaphase-promoting complex/cyclosome, and B4GALT6 contributes to neuronal development and myelin formation (Yoshihara et al., 2018). Taken together, these genes appear to participate in diverse cellular and metabolic processes, prompting further functional enrichment analyses to elucidate their potential roles—especially that of MAN1A2—in gout development.

### 4.2 Potential molecular mechanisms involving MAN1A2 and circMAN1A2

Functional enrichment analyses further explored the biological significance of these DEGs. Gene Ontology and KEGG pathway results revealed enrichment in DNA repair, sphingolipid biosynthesis, and membrane-related processes, predominantly associated with sphingolipid metabolism and the Hedgehog signaling pathway. PPI network analysis suggested potential interactions between MAN1A2 and SOD2. GO annotation indicated that MAN1A2 is mainly involved in protein glycosylation and hydrolytic modification of glycoprotein structures, which are crucial for glycoprotein folding, maturation, trafficking, and biological activity (Tyagi et al., 2023). These findings suggest that MAN1A2 may be associated with biological processes related to cell adhesion, inflammatory responses, and immune regulation. Based on these results, we hypothesize that MAN1A2 may contribute to gout pathogenesis *via* glycosylation-related inflammatory pathways.

To connect the DEG findings with circRNA biology, we further examined circRNA annotations derived from the MAN1A2 locus. Gene ID analysis revealed that a circRNA corresponding to MAN1A2 is hsa_circMAN1A2_011 (chr1:117,944,807–118,009,049; 866 bp). Because circRNAs can be generated from protein-coding gene loci and may participate in post-transcriptional regulation, we selected this MAN1A2 locus-derived circRNA (hereafter referred to as circMAN1A2) for downstream validation.

CircRNAs have been recognized as regulatory molecules acting as microRNA sponges, transcriptional modulators, and protein templates, thereby influencing translation and protein assembly. Bioinformatic prediction showed that hsa-miR-1297, hsa-miR-4465, and hsa-miR-26b-5p potentially bind circMAN1A2, and MAN1A2 was predicted as a potential target gene. Previous studies reported that circMAN1A2 can promote papillary thyroid carcinoma cell proliferation and migration by sponging microRNA-449a and upregulating metadherin expression (Vajen et al., 2022). However, these findings are based solely on computational prediction and should be interpreted with caution. At present, we cannot conclude that circMAN1A2 directly regulates MAN1A2 or gout-related inflammatory pathways through a miRNA sponge mechanism. Future studies employing RNA pull-down or luciferase reporter assays are required to confirm these interactions.

### 4.3 Clinical validation and diagnostic significance

In this study,the bioinformatic discovery phase was limited by the small sample size of the GSE178825 dataset, we mitigated this by implementing a robust validation strategy in a larger, independent clinical cohort (n = 90). The high consistency between transcriptomic predictions and RT-qPCR results confirms circMAN1A2 as a reliable candidate. This sequential ‘discovery-to-validation’ workflow effectively minimizes the risk of false-positive findings inherent in small exploratory screenings.

In recent years, circRNAs have been widely implicated in various physiological and pathological processes. For example, circ-PSD3 has been proposed as a diagnostic biomarker or therapeutic target in papillary thyroid carcinoma (Li et al., 2021; Chen and Shan, 2021),while circFKBP8 and circMBNL1 show aberrant expression in peripheral blood, indicating potential as biomarkers for major depressive disorder (Li et al., 2021; Shi et al., 2021). Nevertheless, studies examining circRNAs as biomarkers for gout remain scarce. Our clinical validation revealed significantly decreased levels of circMAN1A2 in PBMCs from patients with gout compared with healthy individuals. ROC curve analysis demonstrated that circMAN1A2 showed good diagnostic performance with an AUC of 0.86 (95% CI: 0.66–1.00), sensitivity of 90.0%, and specificity of 90.1% in distinguishing gout from controls. High sensitivity is critical for screening assays, enabling timely clinical evaluation. Consequently, circMAN1A2 emerges as a promising blood-based biomarker candidate for gout detection. In addition, the limited (and inverse) discriminative performance of SUA for gout in this cohort may be partly explained by the fluctuation of SUA during acute gout flares (Janssens et al., 2024), during which SUA can be normal or even reduced at presentation, limiting its diagnostic utility in certain clinical contexts.

Correlation analyses demonstrated that circMAN1A2 expression was positively associated with high-density lipoprotein and negatively associated with blood glucose and creatinine levels, suggesting a potential link between circMAN1A2 and metabolic status in gout. Interestingly, MAN1A2 was identified as an upregulated hub gene in the transcriptomic analysis, whereas circMAN1A2 was downregulated in PBMCs from patients with gout. This discordance is not unexpected, as circRNAs and their corresponding linear host-gene transcripts are generated through distinct biogenesis processes. CircRNAs are produced by back-splicing, whereas linear mRNAs are generated by canonical splicing; therefore, their expression can be differentially regulated by splicing factors, RNA-binding proteins, transcriptional activity, and RNA stability. Thus, the opposite expression trends of MAN1A2 and circMAN1A2 do not necessarily indicate a direct regulatory relationship, but rather suggest that circMAN1A2 may be regulated independently of its host gene under gout-related inflammatory or metabolic conditions. Although bioinformatic analysis predicted potential interactions among circMAN1A2, specific miRNAs, and MAN1A2, these findings remain hypothesis-generating. Further mechanistic studies are required to determine whether circMAN1A2 directly regulates MAN1A2 expression and to clarify how this potential interaction may influence gout-related pathways.

Taken together, PBMC-derived circMAN1A2 has practical advantages, including minimally invasive sampling, convenient detection, and potential scalability, supporting its potential utility as a novel blood-based biomarker candidate for gout.

## 5 Limitations and future perspectives

Several limitations should be acknowledged. First, the discovery analysis was based on the small GSE178825 circRNA microarray dataset, which detects back-splice junctions; thus, host-gene annotation was used only for functional interpretation and candidate prioritization, not for inferring linear mRNA expression. However, we compensated for this by validating circMAN1A2 in a much larger, independent clinical cohort, which confirmed its differential expression. Second, our validation cohort included only male participants. This design was adopted to reduce potential confounding effects related to sex hormones, because estrogen levels differ substantially between premenopausal and postmenopausal women and may influence uric acid metabolism, inflammatory responses, and metabolic profiles. Nevertheless, this male-only cohort limits the generalizability of our findings to female patients with gout. Therefore, future multi-center studies with larger, gender-inclusive cohorts are needed, and female participants should be stratified according to menopausal status and, where possible, hormone-related factors. Third, the potential “miRNA sponge” mechanism remains a bioinformatic prediction. While we have discussed potential reasons for the discordant expression between circMAN1A2 and its host gene, further experimental verification (e.g., RNA pull-down) is required. Despite these limitations, this study provides the first evidence for circMAN1A2 as a promising diagnostic biomarker for male patients with gout.

## 6 Conclusion

In summary, this study identified MAN1A2 as a hub gene and its corresponding circRNA, circMAN1A2, as a significantly downregulated molecule in PBMCs from patients with gout. The correlation between circMAN1A2 and metabolic parameters, together with its diagnostic performance, suggests that circMAN1A2 could serve as a novel blood-based biomarker candidate for male patients with gout detection. Future multi-center studies including female cohorts are essential to establish broader clinical utility, while further mechanistic investigations will deepen our understanding of gout pathogenesis and facilitate the development of circRNA-based diagnostic or therapeutic strategies.

## Data Availability

The datasets analysed during the current study are available in the GEO database (GSE178825)(https://www.ncbi.nlm.nih.gov/geo/query/acc.cgi?acc=GSE178825)The initial data for validation of circMAN1A2 in patients with gout during the current study are available from the corresponding author on reasonable request.
