# Supplementary material for: Peripheral blood circMAN1A2 as a novel diagnostic biomarker for gout: integrated transcriptomic analysis and clinical validation
Source: Front Mol Biosci. 2026 May 26;13:1791371. doi: 10.3389/fmolb.2026.1791371 (PMC13246395; doi:10.3389/fmolb.2026.1791371)
Supplement: Supplementary file 1 [file Table1.docx]

| Category | Term | Count | *P* Value | Genes |
| --- | --- | --- | --- | --- |
| CC | cytosol | 27 | 8.22E-05 | USP36, MTPN, SAMD8, CHD9, GSTP1, ANKRD12, SKA3, RPL9, BACH1, FGD4, GPBP1, CPNE1, PIP5K1A, SHOC2, PRKG1, SPECC1, JUN, MBNL1, ANAPC7, NEK6, SMARCA5, TTLL5, PRKD3, APLF, RGS12, PICALM, CSNK1G1 |
| CC | nucleoplasm | 21 | 0.00040114 | SPECC1, USP36, SHPRH, JUN, MBNL1, ANAPC7, CHD9, NONO, NEK6, ANKRD12, SMARCA5, BACH1, SLC8A1, CDC14B, PRKD3, APLF, CPNE1, PIP5K1A, SHOC2, RGS12, RPRD1A |
| CC | nucleus | 24 | 0.005756498 | USP36, CSNK1G3, MTPN, JUN, MBNL1, ANAPC7, SGMS1, GSTP1, NONO, NEK6, SMARCA5, RPL9, BACH1, CDC14B, TTLL5, GPBP1, APLF, CPNE1, PIP5K1A, SHOC2, RGS12, MALAT1, PICALM, CSNK1G1 |
| CC | **membrance** | 13 | 0.014969822 | SPECC1, SGMS1, NONO, RPL9, SLC8A1, RSL1D1, PRKD3, **MAN1A2**, CPNE1, DYM, ME2, PICALM, PCMTD1 |
| CC | RNA Polymerase II transcription factor complex | 3 | 0.034013155 | JUN, NONO, BACH1 |
| CC | Fibrillar center | 3 | 0.049533356 | SPECC1, NONO, SMARCA5 |
| BP | Negative regulation of vascular smooth muscle cell proliferation | 3 | 0.005856712 | GSTP1, SOD2, PRKG1 |
| BP | DNA repair | 5 | 0.006577362 | SHPRH, NONO, SMARCA5, BACH1, CDC14B |
| BP | Sphingolipid biosynthetic process | 3 | 0.006912223 | SAMD8, SGMS1, PRKD3 |
| BP | Hydrogen peroxide reaction | 3 | 0.008047357 | JUN, SOD2, SLC8A1 |
| BP | Response to L-ascorbic acid | 2 | 0.017679957 | GSTP1, SOD2 |
| BP | Sphingomyelin biosynthesis | 2 | 0.027644927 | SAMD8, SGMS1 |
| BP | Regulation of cellular senescence | 2 | 0.030120655 | RSL1D1, NEK6 |
| BP | **Protein glycosylation** | 3 | 0.046314031 | **MAN1A2**, B4GALT6, UGGT2 |
| BP | Response to muscle stretch | 2 | 0.049705441 | JUN, SLC8A1 |
| MF | Nucleosome-dependent ATPase activity | 3 | 0.003257537 | SHPRH, CHD9, SMARCA5 |
| MF | Ceramide cholinephosphotransferase activity | 2 | 0.007477298 | SAMD8, SGMS1 |
| MF | Ceramide phosphoethanolamine synthase activity | 2 | 0.007477298 | SAMD8, SGMS1 |
| MF | Sphingomyelin synthase activity | 2 | 0.007477298 | SAMD8, SGMS1 |
| MF | ATP binding | 10 | 0.013033894 | CSNK1G3, SHPRH, TTLL5, PRKD3, CHD9, NEK6, SMARCA5, PIP5K1A, PRKG1, CSNK1G1 |
| MF | DNA binding | 9 | 0.013485132 | SHPRH, JUN, ZMYM4, CHD9, NONO, GPBP1, SMARCA5, SOD2, BACH1 |

**Table1: The result of GO of DGEs.**

**Table 2A Comparison of datas of patients in three groups (**P* Value vs. control).**

| **Indicator (median)** | **Gout**  **(n= 30)** | **Hyperuricaemia**  **(n = 30)** | **Control**  **(n = 30)** | ***P* Value** |
| --- | --- | --- | --- | --- |
| age (years) | 39.00±3.60 | 34.25±3.95 | 36.75±2.22 | 0.267 |
| TC(mmol/L) | 4.45±1.17 | 5.71±1.03 | 4.56±0.32 | 0.064 |
| TG(mmol/L) | 1.75±1.04* | 1.51±1.03 | 0.90±0.32 | 0.032 |
| LDL(mmol/L) | 3.09±0.84 | 3.70±0.82* | 2.76±0.53 | 0.043 |
| HDL(mmol/L) | 1.00±0.12 | 1.41±0.32* | 1.34±0.03 | 0.003 |
| UA(μmol/L) | 526.20±176.44 | 557.74±139.54* | 378.40±37.98 | 0.006 |
| Cr(μmol/L) | 121.32±15.83* | 87.41±15.97 | 90.09±11.58 | <0.001 |
| Glu(mmol/L) | 5.20±0.55* | 5.18±0.83* | 4.26±0.47 | 0.001 |
| CRP(mg/L) | 18.15±41.81* | 17.36±24.58* | 7.50±0.98 | 0.004 |
| MAN1A2(2^−(ΔΔCt)^) | 0.69±0.31* | 3.95±3.39 | 3.15±2.42 | 0.010 |

Data are presented as mean ± SD. Differences among groups were assessed using one-way ANOVA or Kruskal–Wallis test, as appropriate.

*: *P* Value < 0.05 was considered to denote statistical significance

**Table2B The correlation between each factor and circMAN1A2(**P* Value)**

|  | Total cholesterol  (mmol/L) | Triglyceride  (mmol/L) | Low‐density lipoprotein  (mmol/L) | High‐density lipoprotein  (mmol/L) | Uric acid  (μmol/L) | Creatinine  (μmol/L) | Blood glucose  (mmol/L) | CRP  (mg/L) |
| --- | --- | --- | --- | --- | --- | --- | --- | --- |
| MAN1A2  (2^−(ΔΔCt)^) | -0.189 | -0.244 | -0.286 | 0.526* | -0.216 | -0.484* | -0.400* | -0.363 |
| *P* Value | 0.326 | 0.202 | 0.133 | 0.003 | 0.260 | 0.008 | 0.031 | 0.063 |

| **Table 3 Diagnose efficiency of circMAN1A2 in differentiating the gout or hyperuricemia from normal control.** | | | | | | | | | | |
| --- | --- | --- | --- | --- | --- | --- | --- | --- | --- | --- |
| Test Result Variable(s) | AUC(95%CI) | *P* Value | SD | Cut-off point | Sensitivity  (%) | Specificity  (%) | PPV  (%) | NPV  (%) | LR+ | LR- |
| **Gout group *vs.* Control group** | | | | | | | | |  |  |
| circMAN1A2 | 0.86(0.66,1.00) | 0.006 | 0.10 | 1.22 | 90.0 | 90.1 | 89.0 | 91.0 | 9.89 | 0.11 |
| SUA | 0.28(0.04,0.52) | 0.091 | 0.12 | 276.50 | 100.0 | 9.10 | 50.0 | 100.0 | 1.10 | 0.00 |
| **Hyperuricemia group *vs.* Control group** | | | | | | | | | | |
| circMAN1A2 | 0.49(0.20,0.79) | 0.940 | 0.15 | 5.73 | 44.4 | 90.0 | 80.0 | 64.0 | 3.33 | 1.62 |
| SUA | 0.98(0.92,1.00) | <0.001 | 0.03 | 411.47 | 100.0 | 90.0 | 90.0 | 100.0 | 8.89 | 0.00 |
